# A Comparative Effectiveness of Intravenous Fluids and Insulin Regimens in the Acute Management of Diabetic Ketoacidosis (DKA) and Hypoglycemia: A Systematic Review

**DOI:** 10.7759/cureus.94902

**Published:** 2025-10-19

**Authors:** Samar Mohaen Omar Salem Kanzwl, Ali Hadi M Alhajri, Yousif Jubartalla Abdelbagi Mohammed, Mohamed Abass Ahmed Abdalaziz, Mohammed Alfatih Mohammed Ramadan, Abdulrahman Eltayeb Abdalla Abdelgadir, Gihan Gamaleldeen Abdala Musa

**Affiliations:** 1 Internal Medicine, Medica Hospital, Malé, MDV; 2 Endocrinology, Najran Armed Forces Hospital, Ministry of Defense Health Services, Najran, SAU; 3 Internal Medicine, Maidstone Hospital, Maidstone, GBR; 4 Stroke Medicine, The Royal London Hospital, London, GBR; 5 General Medicine, Bedfordshire Hospitals NHS Foundation Trust, Bedfordshire, GBR; 6 General Medicine, Royal Bolton Hospital, Bolton, GBR; 7 Internal Medicine, Calderdale and Huddersfield NHS Foundation Trust, Huddersfield, GBR

**Keywords:** acute management, diabetic ketoacidosis, hypoglycemia, insulin regimens, intravenous fluids, systematic review

## Abstract

Diabetic ketoacidosis (DKA) and hypoglycemia are acute metabolic emergencies requiring prompt and effective management in both adult and pediatric populations. Despite established protocols, variability in intravenous fluid and insulin regimens persists, necessitating a comprehensive evaluation of their comparative effectiveness across age groups. This systematic review aims to synthesize evidence on the efficacy and safety of different fluid and insulin strategies in managing DKA and hypoglycemia in diverse patient populations.

Following PRISMA 2020 guidelines, a systematic search was conducted across PubMed, Embase, Web of Science, and Scopus. Nine studies (five randomized controlled trials (RCTs) and four cohort studies) were included after screening 227 records. Risk of bias was assessed using Cochrane RoB 2 for RCTs and the Newcastle-Ottawa Scale for cohort studies. Data were synthesized narratively due to clinical heterogeneity.

Early subcutaneous insulin glargine with intravenous insulin reduced DKA resolution time (9.89 ± 3.81 vs. 12.73 ± 5.37 hours; p = 0.022) and hospital stay (4.75 vs. 15.25 days; p = 0.024) compared to intravenous insulin alone. Low-dose insulin (0.05 unit/kg/hour) in pediatric DKA showed comparable efficacy to the standard dose (0.1 unit/kg/hour), with fewer hypoglycemia (3.3% vs. 13.3%) and hypokalemia events (30% vs. 43.3%). Plasmalyte-148 accelerated metabolic acidosis resolution vs. sodium chloride (69% vs. 36% at 24 hours; p = 0.002). For hypoglycemia, 10% and 50% dextrose had similar efficacy, but 50% dextrose required higher doses and caused elevated post-treatment glucose (151.9 vs. 124.6 mg/dL; p = 0.001).

Subcutaneous insulin protocols, low-dose insulin infusions, and balanced crystalloids optimize DKA management, while lower dextrose concentrations may suffice for hypoglycemia. Future research should prioritize multicenter RCTs to validate these findings.

## Introduction and background

Diabetic ketoacidosis (DKA) and hypoglycemia represent two critical metabolic emergencies in patients with diabetes mellitus, each posing significant risks of morbidity and mortality if not promptly and adequately managed [1]. DKA is characterized by absolute or relative insulin deficiency, leading to hyperglycemia, ketonemia, and metabolic acidosis, while hypoglycemia results from excessive insulin action relative to circulating glucose levels, culminating in neuroglycopenic symptoms and potentially life-threatening sequelae [2]. Despite decades of clinical experience, the optimal strategies for acute management of these emergencies continue to evolve, necessitating rigorous evaluation of current intervention protocols to inform evidence-based practice.

The management of DKA is centered on correction of fluid deficits, electrolyte imbalances, and hyperglycemia, traditionally achieved through aggressive intravenous fluid resuscitation and insulin therapy [3]. However, variations exist in the choice of fluid type, infusion rates, and insulin regimens, with implications for patient outcomes, including resolution time, incidence of cerebral edema, electrolyte disturbances, and overall hospital stay [4]. Similarly, treatment of hypoglycemia in emergency settings ranges from oral carbohydrate administration in conscious patients to intravenous dextrose or intramuscular glucagon in severe cases [5]. Yet, heterogeneity in treatment approaches and limited comparative effectiveness data continue to challenge the standardization of protocols across clinical settings.

Recent guidelines provide general recommendations for the use of crystalloids and intravenous insulin in DKA, and for rapid correction of hypoglycemia [6], but emerging studies suggest that tailored approaches - considering patient age, severity, comorbidities, and specific pharmacokinetic properties of interventions - may optimize outcomes [7]. Furthermore, concerns remain regarding risks of fluid overload, hypoglycemia induced by insulin therapy in DKA management, and rebound hyperglycemia in hypoglycemia correction, highlighting the need for a critical synthesis of available evidence [8].

This systematic review aims to comprehensively evaluate and compare the effectiveness of different intravenous fluid regimens and insulin protocols employed in the acute management of DKA and hypoglycemia. By integrating findings from randomized controlled trials (RCTs) and analytical studies, we seek to identify the most efficacious strategies, assess their safety profiles, and delineate gaps in current literature to inform clinical guidelines and future research priorities in acute diabetic emergency care.

## Review

Methodology

Design

This systematic review was conducted in accordance with the Preferred Reporting Items for Systematic Reviews and Meta-Analyses (PRISMA) 2020 guidelines [9] to ensure transparency and methodological rigor.

Eligibility Criteria

We included original studies that evaluated the comparative effectiveness of different intravenous fluid regimens and insulin protocols in the acute management of DKA or hypoglycemia. Eligible studies comprised RCTs, non-randomized controlled studies, and observational cohort studies published in English. Studies conducted on adult or pediatric populations presenting with DKA or hypoglycemia in emergency, critical care, or inpatient settings were included. We excluded case reports, case series with fewer than 10 patients, editorials, conference abstracts without full texts, and narrative or systematic reviews.

Information Sources and Search Strategy

A comprehensive literature search was conducted across four electronic databases: PubMed, Embase, Web of Science, and Scopus. The search strategy was designed in consultation with an experienced medical librarian to maximize sensitivity and included a combination of Medical Subject Headings (MeSH) and free-text terms related to “diabetic ketoacidosis,” “hypoglycemia,” “intravenous fluids,” “insulin regimens,” and “treatment outcomes.” Additionally, the reference lists of included studies and relevant reviews were manually screened to identify any additional eligible studies. The detailed search strings and search dates for each database are provided in Table 1. 

**Table 1:** Search Strategy and Search Dates for All Databases

Database	Search Dates	Search Strategy/Search String	Filters Applied
PubMed (MEDLINE)	August 27, 2025	("Diabetic Ketoacidosis"[Mesh] OR "DKA" OR "diabetic ketoacidosis" OR "ketoacidosis, diabetic") AND ("Hypoglycemia"[Mesh] OR "hypoglycemia" OR "low blood glucose") AND ("Insulin"[Mesh] OR "insulin therapy" OR "insulin regimen" OR "intravenous insulin") AND ("Fluid Therapy"[Mesh] OR "intravenous fluids" OR "fluid replacement" OR "rehydration") AND ("Randomized Controlled Trial"[Publication Type] OR "Cohort Studies"[Mesh] OR "Clinical Study")	Humans; English language
Embase	September 1, 2025	('diabetic ketoacidosis'/exp OR 'DKA' OR 'diabetic ketoacidosis') AND ('hypoglycemia'/exp OR 'hypoglycemia' OR 'low blood glucose') AND ('insulin therapy'/exp OR 'insulin infusion' OR 'insulin regimen') AND ('fluid therapy'/exp OR 'intravenous fluids' OR 'rehydration') AND ([randomized controlled trial]/lim OR [cohort]/lim OR [clinical study]/lim)	Humans; English language
Web of Science (Core Collection)	September 1, 2025	TS=("diabetic ketoacidosis" OR "DKA") AND TS=("hypoglycemia" OR "low blood sugar") AND TS=("insulin regimen" OR "intravenous insulin" OR "insulin therapy") AND TS=("fluid therapy" OR "intravenous fluids" OR "rehydration")	Document type: Article; English language
Scopus	September 2, 2025	TITLE-ABS-KEY("diabetic ketoacidosis" OR "DKA") AND TITLE-ABS-KEY("hypoglycemia" OR "low blood glucose") AND TITLE-ABS-KEY("insulin regimen" OR "intravenous insulin" OR "insulin therapy") AND TITLE-ABS-KEY("fluid therapy" OR "intravenous fluids" OR "rehydration") AND (LIMIT-TO(DOCTYPE, "ar"))	Articles only; English language

Study Selection

All identified records were imported into EndNote X9 (Clarivate, Philadelphia, PA, USA) for reference management, and duplicates were removed. Two reviewers independently screened titles and abstracts for relevance based on predefined eligibility criteria. Full texts of potentially eligible studies were retrieved and assessed independently by the same reviewers. Discrepancies at any stage were resolved through discussion or consultation with a third reviewer to achieve consensus.

Data Extraction

Data extraction was performed independently by two reviewers using a standardized data extraction form developed for this review. Extracted data included study characteristics (first author, year of publication, country, setting, and study design), population characteristics (sample size, age, sex, and clinical condition), intervention details (type of intravenous fluid, insulin regimen, dosage, and administration protocol), comparator interventions, duration of follow-up, outcomes assessed (including resolution time, adverse events, mortality, and hospital length of stay), and key findings. Any discrepancies were resolved through discussion and consensus.

Risk of Bias Assessment

The risk of bias for included RCTs was assessed using the revised Cochrane risk-of-bias tool for randomized trials (RoB 2) [10], evaluating domains such as randomization process, deviations from intended interventions, missing outcome data, measurement of the outcome, and selection of the reported result. For cohort studies, the Newcastle-Ottawa Scale (NOS) [11] was employed to assess methodological quality across selection, comparability, and outcome domains. Two reviewers independently performed the assessments, and disagreements were resolved through discussion.

Data Synthesis

A narrative synthesis of findings was conducted, structured around the type of intervention, comparator, outcomes assessed, and study design. Where appropriate, data were tabulated to allow comparison across studies. Given the clinical and methodological heterogeneity, quantitative synthesis through meta-analysis was not considered.

Results

Search Results

**Figure 1:**
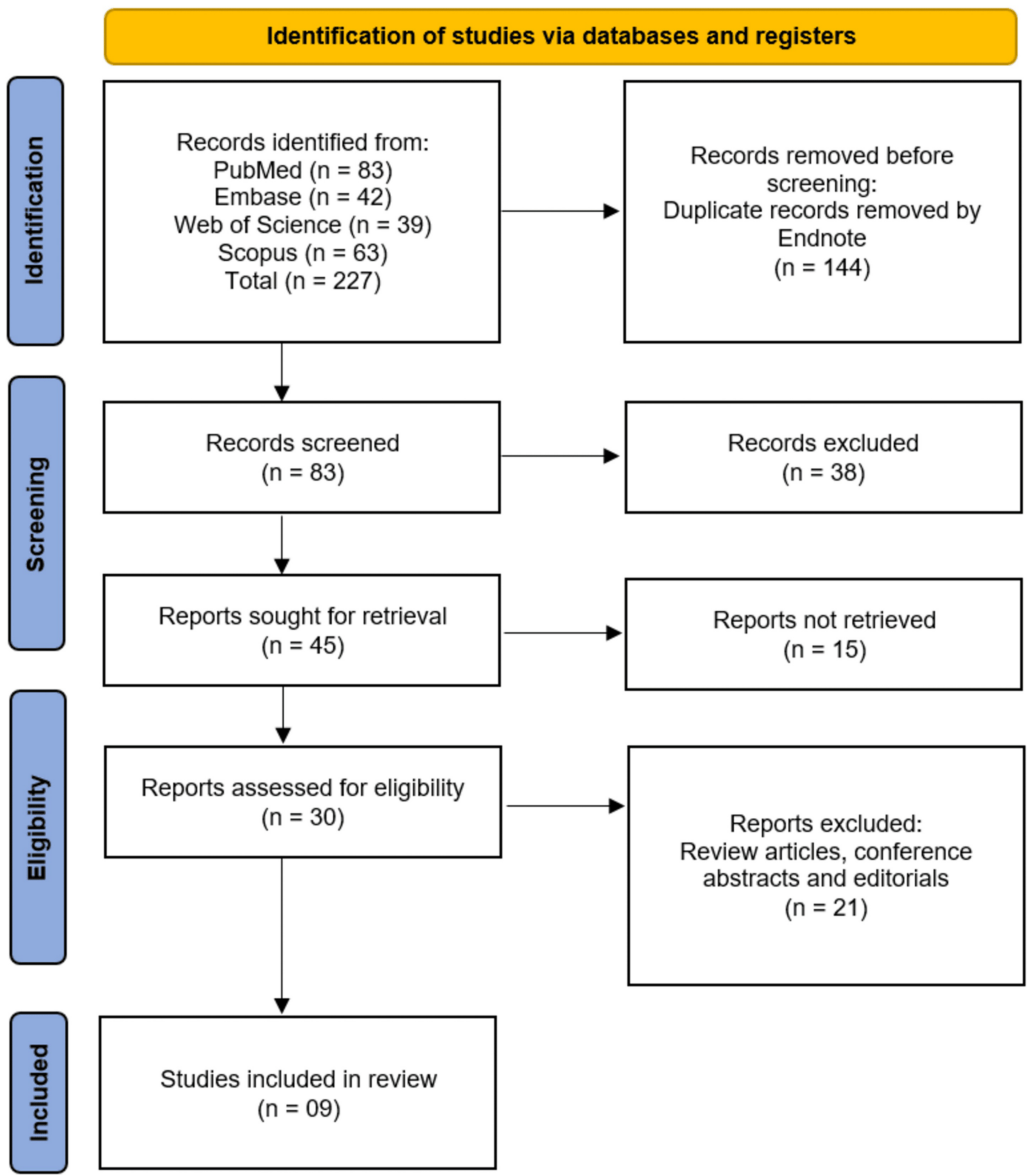
PRISMA Diagram Indicating the Study Selection Process This diagram shows the process of identifying, screening, and selecting studies for inclusion in the systematic review. Out of 227 records from four databases, 144 duplicates were removed. After screening 83 titles and abstracts, 45 full-text articles were assessed, and nine studies met eligibility criteria. Reasons for exclusion at each stage are provided, following PRISMA 2020 guidelines.

The systematic review followed the PRISMA guidelines for study selection (Figure 1). A total of 227 records were initially identified through database searches, including PubMed (n = 83), Embase (n = 42), Web of Science (n = 39), and Scopus (n = 63). After removing 144 duplicate records, 83 studies underwent title and abstract screening. Of these, 38 records were excluded as irrelevant. The remaining 45 full-text articles were sought for retrieval, of which 15 could not be accessed. A total of 30 studies were assessed for eligibility, and 21 were excluded for being review articles, conference abstracts, or editorials. Ultimately, nine studies met the inclusion criteria and were included in the systematic review [12-20].

Study Characteristics

The systematic review included nine studies examining the comparative effectiveness of intravenous fluids and insulin regimens in the acute management of DKA and hypoglycemia [12-20]. The studies were conducted across diverse geographical settings, including Thailand, Egypt, India, Australia, Japan, and the United States, and encompassed both adult and pediatric populations (Table 2). Study designs varied, with five RCTs [12-16], three retrospective cohort studies [17-19], and one retrospective cohort study focusing on prehospital emergency medical services (EMS) settings [20]. The sample sizes ranged from 60 participants in a single-center RCT [12] to 14,216 inpatients in a nationwide cohort study [18]. The majority of studies evaluated DKA management, while two focused on hypoglycemia treatment [14,20].

**Table 2:** General Characteristics of Included Studies This table summarizes the key features of the nine studies included in the review. Information presented includes study setting, design, population characteristics, sample size, and condition studied (DKA or hypoglycemia). The table highlights the diversity of geographical locations, patient age groups (adult and pediatric), and study designs (RCTs and cohort studies). DKA, Diabetic Ketoacidosis; RCT, Randomized Controlled Trial; PL, Plasmalyte; SC, Sodium Chloride; EMS, Emergency Medical Services; D10, 10% Dextrose; D50, 50% Dextrose

Study (Year)	Country/Setting	Study Design	Population Characteristics	Condition Studied
Thammakosol and Sriphrapradang (2023) [12]	Thailand/Single-center study	Single-center, open-label RCT	Adults ≥18 years with DKA (n = 60; mostly type 2 diabetes, 76.7%)	DKA
Hawary et al. (2025) [13]	Egypt	RCT	100 pediatric patients with DKA (50 per group)	DKA
Verma et al. (2024) [14]	India/Urban tertiary care center	Randomized controlled single-blinded study	Hypoglycemic patients in altered mental status; total analyzed n = 204	Hypoglycemia and DKA
Ramanan et al. (2021) [15]	Australia/Seven hospitals	Cluster, crossover, open-label, randomized, controlled phase 2 trial	Adults admitted to ICU with severe DKA; 93 enrolled (PL n = 48, SC n = 42)	DKA
Nallasamy et al. (2014) [16]	India	Randomized, double-blind controlled clinical trial	Children aged ≤12 years with DKA; excluded if septic shock or prior insulin	Pediatric DKA
Goad et al. (2020) [17]	USA/Hospital setting	Retrospective cohort study	102 adult patients admitted with incident DKA from January 2013 to October 2017	DKA
Okada et al. (2021) [18]	Japan/Japanese Diagnosis Procedure Combination database; nationwide inpatient setting	Retrospective cohort study	14,216 inpatients with diabetic ketoacidosis admitted between July 2010 and March 2018; excluded patients with kidney dysfunction or serum potassium abnormalities	DKA
Rao et al. (2022) [19]	USA/Integrated health care system in Northern California (21 hospitals)	Retrospective cohort study evaluating a prospectively implemented protocol	7989 hospitalizations for diabetic ketoacidosis; mean age 42.3 years (SD 17.7); 51.8% female	DKA
Weant et al. (2021) [20]	USA/Prehospital EMS setting (patients transported to an academic teaching hospital emergency department)	Retrospective cohort study	478 patients with hypoglycemia treated by EMS between 2014 and 2017; 161 received D10 and 150 received D50	Hypoglycemia

Interventions and Comparators

The interventions and comparators across the studies were heterogeneous, reflecting diverse clinical approaches to DKA and hypoglycemia management (Table 3). For DKA, key interventions included early subcutaneous insulin glargine combined with intravenous insulin [12], concurrent long-acting insulin with intravenous insulin [13], low-dose versus standard-dose insulin infusions [16], and the use of Plasmalyte-148 versus sodium chloride for fluid therapy [15]. Subcutaneous insulin protocols were also compared to traditional intravenous insulin regimens [19]. For hypoglycemia, studies compared different concentrations of dextrose (10%, 25%, and 50%) in emergency and prehospital settings [14,20].

**Table 3:** Intervention, Comparator, Outcomes, and Findings This table details the specific interventions and comparators evaluated in each study, the outcomes assessed, and the main findings. It also notes the duration of follow-up and adverse events, where available. The data allow side-by-side comparison of different insulin regimens, fluid therapies, and dextrose concentrations used for managing DKA and hypoglycemia. DKA, Diabetic Ketoacidosis; RCT, Randomized Controlled Trial; PL, Plasmalyte; SC, Sodium Chloride; EMS, Emergency Medical Services; GCS, Glasgow Coma Scale; AKI, Acute Kidney Injury; NR, Not Reported; LOS, Length of Stay; D10, 10% Dextrose; D50, 50% Dextrose

Study (Year)	Intervention	Comparator	Outcomes Assessed	Duration of Follow-Up	Key Findings	Adverse Events
Thammakosol and Sriphrapradang (2023) [12]	Early combination of subcutaneous insulin glargine (0.3 units/kg within the first 3 hours) with IV insulin infusion	IV insulin infusion alone	Primary: Time to DKA resolution; Secondary: Rebound hyperglycemia, mortality, hypoglycemia, hypokalaemia, LOS	Until DKA resolution and hospital discharge (LOS measured in days)	Early glargine group had significantly faster DKA resolution (9.89 ± 3.81 vs. 12.73 ± 5.37 hours; p = 0.022) and shorter LOS (median 4.75 vs. 15.25 days; p = 0.024) compared to control.	Incidence of rebound hyperglycemia, mortality, hypoglycemia, and hypokalemia was similar between groups.
Hawary et al. (2025) [13]	Concurrent administration of subcutaneous long-acting insulin alongside intravenous insulin during DKA treatment	Traditional DKA management protocol (intravenous insulin alone)	Duration of insulin infusion, insulin dose required, hypoglycemia events, hypokalemia events	During hospital DKA treatment (median infusion duration reported up to ~72 hours)	Coadministration of subcutaneous long-acting insulin reduced duration (68.5 vs 72 hours, p = 0.0001) and dose of insulin infusion (3.48 vs 4.04 units/kg, p = 0.016). It also decreased hypoglycemia events (12 vs 22, p = 0.029) without increasing hypokalemia risk (19 vs 12, p = 0.147).	Reduced hypoglycemia events in intervention group; no increase in hypokalemia events.
Verma et al. (2024) [14]	5 g boluses of 10% dextrose	5 g boluses of 25% and 50% dextrose	Time to achieve GCS 15; Median total dose administered	Until the achievement of GCS 15 (median ~6 minutes)	No difference in median time to achieve GCS 15 across groups (all ~6 min). The total median dose was lower in the 10% and 25% groups (10 g) compared to 50% group (15 g). Higher proportion of patients received the maximum dose (25 g) in 50% group (12%) vs 10% (3%) and 25% (4%).	NR
Ramanan et al. (2021) [15]	PL-148 intravenous fluid therapy	0.9% SC intravenous fluid therapy	DKA resolution (base excess ≥ -3 mEq/L), Anion gap, Blood ketones, ICU length of stay, Hospital length of stay	48 hours (primary outcome assessed at 48 h; additional data at 24 h)	DKA resolution at 48 h: 96% (PL) vs 86% (SC), OR 3.93 (95% CI 0.73-21.16, p = 0.111); DKA resolution at 24 h: 69% (PL) vs 36% (SC), OR 4.24 (95% CI 1.68-10.72, p = 0.002); Median anion gap and blood ketones similar between groups; Median ICU stay: 49 h (PL) vs 55 h (SC); Median hospital stay: 81 h (PL) vs 98 h (SC). Conclusion: PL may lead to faster resolution of metabolic acidosis without increasing ketosis.	NR
Nallasamy et al. (2014) [16]	Low-dose insulin infusion (0.05 Unit/kg/hour)	Standard-dose insulin infusion (0.1 Unit/kg/hour)	Primary: Time for resolution of DKA (pH ≥7.3, bicarbonate ≥15 mEq/L, beta-hydroxybutyrate <1 mmol/L); Secondary: Rate of fall in blood glucose until ≤250 mg/dL, rate of complications (hypokalemia, hypoglycemia, cerebral edema)	Up to resolution of DKA (mean ~22-23 hours)	Time for resolution was similar between groups. Low-dose group had a lower adjusted hazard ratio for resolution (0.40; p = 0.017). Rates of glucose fall and time to achieve target were similar.	Hypokalemia: 30% (low-dose) vs 43.3% (standard-dose); Hypoglycemia: 3.3% (low-dose) vs 13.3% (standard-dose); No cerebral edema or mortality in either group.
Goad et al. (2020) [17]	Development of hyperchloremia during DKA management	Maintaining normochloremia during DKA management	Primary: Time to final DKA resolution; Secondary: Time to initial DKA resolution, incidence of AKI on admission, in-hospital development of AKI, hospital LOS	In-hospital stay (median time to resolution measured in hours)	Hyperchloremia was associated with: longer time to final DKA resolution (median 22.3 vs. 14.2 hours; p = 0.001); longer time to initial DKA resolution (median 16.3 vs. 10.9 hours; p = 0.024); increased in-hospital AKI (26.9% vs. 8.0%; p = 0.01); and longer hospital LOS (p < 0.001; each mmol increase in chloride prolonged DKA resolution).	Increased incidence of in-hospital acute kidney injury (26.9% vs 8.0%).
Okada et al. (2021) [18]	Potassium replacement at different concentrations (within initial fluid therapy; specifically concentrations ranging approximately from 10 to 40 mmol/L)	Lower potassium concentrations (<10 mmol/L)	28-day in-hospital mortality; occurrence of hyperkalemia	28 days (first 2 days potassium infusion assessed; 28-day mortality follow-up)	Potassium concentrations of 10-40 mmol/L were not associated with increased mortality or hyperkalemia. However, lower potassium concentrations were associated with higher 28-day in-hospital mortality (e.g., OR 1.69 for 8 mmol/L vs. 20 mmol/L).	No significant difference in the occurrence of hyperkalemia across potassium concentration groups.
Rao et al. (2022) [19]	Subcutaneous insulin treatment protocol for diabetic ketoacidosis	Standard care with intravenous insulin	ICU admission, 30-day hospital readmission, hospital length of stay, mortality	Preimplementation phase: 2010-2015; Postimplementation phase: 2017-2019	Subcutaneous insulin protocol was associated with a 57% reduction in ICU admissions (adjusted rate ratio 0.43, 95% CI 0.33-0.56) and 50% reduction in 30-day hospital readmissions (adjusted rate ratio 0.50, 95% CI 0.25-0.99). No significant change in hospital length of stay or mortality.	No evidence of increases in adverse events reported.
Weant et al. (2021) [20]	10% dextrose (D10) IV administration by EMS	50% dextrose (D50) IV administration by EMS	Need for dextrose retreatment prior to hospital arrival; Prehospital reassessment glucose; Glucose on hospital arrival; Hospital admission; Length of stay; In-hospital mortality	Until hospital arrival and in-hospital outcomes	No significant difference in need for retreatment before hospital arrival (0.6% D10 vs 2.0% D50; p = 0.565); D50 group had significantly higher prehospital glucose (151.9 vs 124.6 mg/dL, p = 0.001) and arrival glucose (129.5 vs 108.0 mg/dL, p = 0.011); No difference in hospital admission, length of stay, or in-hospital mortality.	NR

Outcomes and Key Findings

DKA management: Early administration of subcutaneous insulin glargine alongside intravenous insulin significantly reduced the time to DKA resolution (9.89 ± 3.81 vs. 12.73 ± 5.37 hours; p = 0.022) and shortened hospital length of stay (median 4.75 vs. 15.25 days; p = 0.024) compared to intravenous insulin alone [12]. Similarly, the coadministration of long-acting insulin with intravenous insulin in pediatric patients decreased the duration of insulin infusion (68.5 vs. 72 hours; p = 0.0001) and reduced hypoglycemia events (12 vs. 22; p = 0.029) without increasing hypokalemia risk [13]. Low-dose insulin infusion (0.05 units/kg/hour) demonstrated comparable efficacy to standard-dose (0.1 units/kg/hour) in resolving pediatric DKA, but with lower rates of hypoglycemia (3.3% vs. 13.3%) and hypokalemia (30% vs. 43.3%) [16].

Fluid therapy with Plasmalyte-148 showed a trend toward faster DKA resolution at 24 hours (69% vs. 36%; OR 4.24, 95% CI 1.68-10.72; p = 0.002) compared to sodium chloride, though the difference at 48 hours was not statistically significant [15]. Hyperchloremia during DKA management was associated with prolonged time to resolution (median 22.3 vs. 14.2 hours; p = 0.001) and increased acute kidney injury (AKI) (26.9% vs. 8.0%; p = 0.01). Subcutaneous insulin protocols reduced ICU admissions by 57% (adjusted rate ratio 0.43, 95% CI 0.33-0.56) and 30-day readmissions by 50% (adjusted rate ratio 0.50, 95% CI 0.25-0.99) without affecting mortality or hospital length of stay [19].

Hypoglycemia management: In the emergency department, no significant differences were observed in the time to achieve Glasgow Coma Scale (GCS) 15 across dextrose concentrations (10%, 25%, and 50%), though the 50% dextrose group required a higher median total dose (15 g vs. 10 g) [14]. In prehospital settings, 10% dextrose (D10) and 50% dextrose (D50) showed no difference in retreatment needs or hospital outcomes, though D50 resulted in higher post-treatment glucose levels (151.9 vs. 124.6 mg/dL; p = 0.001) [20].

Adverse Events

Adverse events were inconsistently reported across studies. For DKA, hypoglycemia and hypokalemia were the most common complications, with lower-dose insulin regimens associated with reduced risks [12,13,16]. Hyperchloremia was linked to unfavorable outcomes, including AKI [17]. No significant differences in hyperkalemia were observed with varying potassium replacement strategies [18]. In hypoglycemia studies, adverse events were rarely reported, though higher dextrose concentrations did not increase morbidity [14,20].

Risk of Bias Assessment

The risk of bias for the five RCTs was assessed using the Cochrane RoB 2 tool (Table 4). Two studies - Verma et al. [14] and Nallasamy et al. [16] - were rated as low risk across all domains, including randomization, deviations, missing data, outcome measurement, and reporting. The remaining RCTs - Thammakosol and Sriphrapradang [12], Hawary et al. [13], and Ramanan et al. [15] - had some concerns, primarily due to lack of blinding (open-label designs) or potential imbalances in cluster randomization [15]. No studies were rated as high risk.

**Table 4:** Risk of Bias Assessment Using Cochrane RoB 2 Tool for RCTs This table presents the risk of bias assessment for the five included RCTs. It evaluates key domains: randomization, deviations from intended interventions, missing outcome data, outcome measurement, and reporting bias. The overall judgment is provided for each study, indicating whether the trial was rated as low risk or raised some concerns. RCT, Randomized Controlled Trial

Study (Year)	Randomization Process	Deviations From Intended Interventions	Missing Outcome Data	Measurement of Outcomes	Selection of Reported Results	Overall Risk of Bias
Thammakosol and Sriphrapradang (2023) [12]	Low	Some concerns	Low	Low	Low	Some concerns
Hawary et al. (2025) [13]	Low	Some concerns	Low	Low	Low	Some concerns
Verma et al. (2024) [14]	Low	Low	Low	Low	Low	Low
Ramanan et al. (2021) [15]	Some concerns	Some concerns	Low	Low	Low	Some concerns
Nallasamy et al. (2014) [16]	Low	Low	Low	Low	Low	Low

For the four cohort studies, the NOS was applied (Table 5). Okada et al. [18] achieved the highest score (9/9), reflecting a well-defined nationwide cohort, robust adjustment for confounders, and objective outcome assessment. Goad et al. [17] and Rao et al. [19] also scored well (8/9), with minor limitations in cohort representativeness. Weant et al. [20] had a slightly lower score (7/9) due to limited adjustment for confounders but maintained low risk overall. No cohort studies were rated as high risk.

**Table 5:** Risk of Bias Assessment Using Newcastle-Ottawa Scale (NOS) for Cohort Studies This table reports the methodological quality of the four included cohort studies using the NOS. Scores are shown for three domains: selection of participants, comparability of cohorts, and outcome assessment. Total scores (out of 9) are listed, along with overall risk-of-bias classification. Most studies scored high, indicating good quality with low risk of bias.

Study (Year)	Selection (Max 4)	Comparability (Max 2)	Outcome (Max 3)	Total Score (Max 9)	Risk of Bias
Goad et al. (2020) [17]	3	2	3	8	Low
Okada et al. (2021) [18]	4	2	3	9	Low
Rao et al. (2022) [19]	3	2	3	8	Low
Weant et al. (2021) [20]	3	1	3	7	Low

Discussion

The findings of this systematic review provide a comprehensive synthesis of the comparative effectiveness of intravenous fluids and insulin regimens in the acute management of DKA and hypoglycemia. The included studies, spanning diverse geographical settings and patient populations, highlight several key trends and clinical implications. Notably, the integration of subcutaneous insulin with intravenous insulin emerged as a promising strategy for DKA management, demonstrating faster resolution times and reduced hospital stays compared to traditional intravenous insulin alone. For instance, Thammakosol and Sriphrapradang [12] reported that early administration of insulin glargine, alongside intravenous insulin, significantly shortened the time to DKA resolution (9.89 ± 3.81 vs. 12.73 ± 5.37 hours) and reduced hospital length of stay (median 4.75 vs. 15.25 days). These findings align with prior research advocating for early transition to subcutaneous insulin to mitigate the risks of prolonged intravenous therapy, such as hypoglycemia and hypokalemia [21]. Similarly, Hawary et al. [13] observed that coadministration of long-acting insulin with intravenous insulin in pediatric patients not only reduced the duration of insulin infusion, but also decreased hypoglycemia events, reinforcing the potential benefits of this approach in younger populations.

The efficacy of low-dose insulin infusions (0.05 unit/kg/hour) in pediatric DKA, as demonstrated by Nallasamy et al. [16], challenges the conventional use of higher doses (0.1 unit/kg/hour). The study reported comparable resolution times but significantly lower rates of hypoglycemia (3.3% vs. 13.3%) and hypokalemia (30% vs. 43.3%), suggesting that lower doses may offer a safer alternative without compromising therapeutic outcomes. This is particularly relevant given the heightened vulnerability of pediatric patients to complications like cerebral edema [22]. The findings echo those of Edge et al. [23], who also reported no significant differences in DKA resolution between low- and standard-dose insulin regimens, further supporting the adoption of lower doses in clinical practice.

Fluid therapy choices in DKA management also warrant careful consideration. Ramanan et al. [15] found that Plasmalyte-148, a balanced crystalloid, was associated with faster metabolic acidosis resolution at 24 hours compared to sodium chloride (69% vs. 36%), though the difference at 48 hours was not statistically significant. This aligns with growing evidence favoring balanced solutions over normal saline in critical care settings, as hyperchloremia from saline has been linked to adverse outcomes, including AKI [24]. The study by Goad et al. [17] corroborates this, showing that hyperchloremia prolonged DKA resolution (median 22.3 vs. 14.2 hours) and increased AKI incidence (26.9% vs. 8.0%). These findings underscore the importance of selecting physiologically balanced fluids to optimize patient outcomes, a recommendation increasingly reflected in recent guidelines [25].

Subcutaneous insulin protocols for DKA, as evaluated by Rao et al. [19], demonstrated significant reductions in ICU admissions (57%) and 30-day readmissions (50%) without affecting mortality or hospital length of stay. This suggests that subcutaneous insulin may be a viable alternative to intravenous insulin in select patients, particularly in resource-limited settings where ICU beds are scarce. However, the applicability of these protocols may depend on patient severity, as those with severe DKA or altered mental status may still require intensive monitoring. The results are consistent with earlier studies by Umpierrez et al. [26], who reported successful DKA management with subcutaneous insulin in mild to moderate cases, though further research is needed to validate these findings in broader populations.

In the context of hypoglycemia management, the studies by Verma et al. [14] and Weant et al. [20] provide valuable insights into dextrose concentration selection. While no differences were observed in time to GCS recovery across 10%, 25%, and 50% dextrose groups, the higher concentration (50%) was associated with greater total dextrose requirements and elevated post-treatment glucose levels. This raises concerns about potential overtreatment and rebound hyperglycemia, particularly in prehospital settings where monitoring is limited. These findings resonate with those of Moore and Woollard [27], who cautioned against excessive dextrose administration due to its association with adverse glycemic variability. The lack of significant differences in retreatment needs between 10% and 50% dextrose, as reported by Weant et al. [20], further supports the use of lower concentrations, which may offer a safer profile without compromising efficacy.

Adverse events were inconsistently reported across studies, but hypoglycemia and hypokalemia emerged as the most common complications in DKA management. The lower incidence of these events with low-dose insulin and subcutaneous protocols highlights the need for tailored insulin regimens to minimize risks. Hyperchloremia’s association with AKI, as noted by Goad et al. [17], reinforces the importance of fluid selection, while the absence of hyperkalemia with varying potassium replacement strategies suggests that moderate potassium supplementation is safe [18]. For hypoglycemia, the limited reporting of adverse events precludes definitive conclusions, though the higher dextrose concentrations did not appear to increase morbidity.

The risk of bias assessment revealed generally robust methodologies, with most RCTs and cohort studies, rated as low or moderate risk. However, the open-label designs of some RCTs introduced performance bias, as blinding was not feasible. This limitation is common in DKA trials due to the pragmatic challenges of masking insulin therapies [28]. The cohort studies, particularly Okada et al. [18] and Rao et al. [19], demonstrated high methodological quality, though residual confounding remains a potential issue in observational designs.

Limitations

Despite its strengths, this review has several limitations. First, the heterogeneity in study designs, populations, and interventions precluded meta-analysis; limiting the ability to quantify pooled effects. Second, the predominance of single-center RCTs and retrospective cohort studies may affect generalizability; as findings from specialized settings may not translate to broader clinical practice. Third, the exclusion of non-English studies and unpublished data could introduce publication bias. Finally, the varying definitions of outcomes (e.g., DKA resolution) across studies complicate direct comparisons.

## Conclusions

This systematic review underscores the evolving landscape of DKA and hypoglycemia management, with evidence supporting the use of subcutaneous insulin, low-dose insulin infusions, and balanced crystalloids to improve outcomes and reduce complications. The findings advocate for personalized treatment approaches, tailored to patient severity and clinical context. Future research should prioritize large, multicenter RCTs to validate these strategies and address existing gaps, particularly in pediatric and prehospital settings. Until then, clinicians are encouraged to integrate these evidence-based practices into their protocols, balancing efficacy with safety to optimize patient care.
